# Frequent occurrence of *Mungbean yellow mosaic India virus* in tomato leaf curl disease affected tomato in Oman

**DOI:** 10.1038/s41598-019-53106-4

**Published:** 2019-11-12

**Authors:** M. S. Shahid, M. Shafiq, M. Ilyas, A. Raza, M. N. Al-Sadrani, A. M. Al-Sadi, R. W. Briddon

**Affiliations:** 1 Department of Crop Sciences, College of Agricultural and Marine Sciences, Sultan Qaboos University, Al-Khod, 123 Oman; 2 Cell Biology and Molecular Genetics, University of Maryland College Park, MD, 20742 USA; 3 Agricultural Biotechnology Division, National Institute for Biotechnology and Genetic Engineering, Faisalabad, Pakistan

**Keywords:** Biotechnology, Plant molecular biology

## Abstract

Next generation sequencing (NGS) of DNAs amplified by rolling circle amplification from 6 tomato (*Solanum lycopersicum*) plants with leaf curl symptoms identified a number of monopartite begomoviruses, including *Tomato yellow leaf curl virus* (TYLCV), and a betasatellite (*Tomato leaf curl betasatellite* [ToLCB]). Both TYLCV and ToLCB have previously been identified infecting tomato in Oman. Surprisingly the NGS results also suggested the presence of the bipartite, legume-adapted begomovirus *Mungbean yellow mosaic Indian virus* (MYMIV). The presence of MYMIV was confirmed by cloning and Sanger sequencing from four of the six plants. A wider analysis by PCR showed MYMIV infection of tomato in Oman to be widespread. Inoculation of plants with full-length clones showed the host range of MYMIV not to extend to *Nicotiana benthamiana* or tomato. Inoculation to *N. benthamiana* showed TYLCV to be capable of maintaining MYMIV in both the presence and absence of the betasatellite. In tomato MYMIV was only maintained by TYLCV in the presence of the betasatellite and then only at low titre and efficiency. This is the first identification of TYLCV with ToLCB and the legume adapted bipartite begomovirus MYMIV co-infecting tomato. This finding has far reaching implications. TYLCV has spread around the World from its origins in the Mediterranean/Middle East, in some instances, in live tomato planting material. The results here may suggest that begomoviruses which do not commonly infect tomato, such as MYMIV, could be spread as a passenger of TYLCV in tomato.

## Introduction

Cultivation of tomato in Oman has in recent years suffered serious losses due to tomato leaf curl disease (ToLCD). ToLCD is caused by begomoviruses; viruses of the genus *Begomovirus* (family *Geminiviridae*)^1^. To date five distinct begomovirus species have been identified in tomato with ToLCD symptoms in the country, including *Tomato yellow leaf curl virus* (TYLCV)^2^. TYLCV was initially identified in the Middle East in the 1960s and has since spread to most tropical and sub-tropical regions of the world^3,4^.

Viruses with circular single-stranded (ss) DNA genomes encapsidated in paired quasi-icosahedral capsids are classified in the family *Geminiviridae*. Geminiviruses are transmitted plant-to-plant by specific arthropod vectors. The family comprises of nine genera^1^, of which the most widespread and most destructive are viruses of the genus *Begomovirus* that are transmitted by the whitefly *Bemisia tabaci*^5^. The New World (NW) native begomoviruses typically have two-component genomes (components DNA A and DNA B), whereas most native to the Old World (OW) have monopartite genomes (a homolog of the DNA A of bipartite viruses). Few bipartite begomoviruses have been identified in the OW but only three monopartite begomoviruses have so far been identified in the NW^6–8^.

The genomes of monopartite begomoviruses, and their bipartite begomovirus homolog DNA A, originating from the OW encode six genes. Virion-sense strand transcribed genes encode the coat protein (CP) and V2 protein. The complementary-sense strand codes for four genes. These encode the replication-associated protein (Rep), the transcriptional-activator protein (TrAP), the replication enhancer protein (REn) and the C4 protein^9,10^. Bipartite begomovirus DNA B components encode one gene on the complementary-sense, the movement protein (MP), and one on the virion-sense, the nuclear shuttle protein (NSP), which have an essential role in virus intra- and intercellular movement in plants^11^.

Generally OW monopartite begomoviruses associate with small (1.3–1.4 kb) circular ssDNA molecules referred to as alphasatellites and betasatellites. Alphasatellites (previously known as DNA 1^12^) encode a Rep (a rolling-circle replication initiator protein) and replicate autonomously of the helper virus. Satellites, by definition, are reliant on a helper virus for replication and alphasatellites must thus be described as satellite-like^13^. Although not essential for the infectivity of the helper virus, alphasatellites may enhance or attenuate virus symptoms in plants and rely upon helper viruses for movement *in planta* as well as plant-to-plant transmission. The Rep encoded by alphasatellites has been shown to suppress RNA silencing^14,15^. In contrast betasatellites (previously known as DNA β) rely on helper viruses for replication, movement in plants and transmission. Betasatellites may enhance helper virus induced symptoms as well as raising viral DNA levels in plants. The structure of betasatellite is highly conserved, encompassing a sequence that is highly conserved among betasatellites (the satellite conserved region [SCR]), a sequence rich in adenine residues (A-rich) and a single, complementary-sense strand encoded, gene known as βC1^16^. The βC1 protein is a dominant pathogenicity determinant, may have a role in virus movement in plants and has suppressor of RNA silencing activity^17^.

Five distinct begomovirus species have been identified that affecting tomato cultivation in Oman (reviewed by Khan *et al*.^18^). These include viruses which are believed to have been introduced into Oman, TYLCV^19^, *Chili leaf curl virus* (ChiLCV)^20^ and *Tomato leaf curl Sudan virus* (ToLCSDV)^21^, as well as begomoviruses so far identified only in Oman, *Tomato leaf curl Liwa virus* (ToLCLwV, previously known as *Tomato leaf curl Al-Batinah virus*)^22^ and *Tomato leaf curl Barka virus* (ToLCBrV)^23^. Only two betasatellites have been identified in Oman, the most important of which is *Tomato leaf curl betasatellite* (ToLCB), which has its origin on the Indian sub-continent^18^. ToLCB in Oman has been shown to occur in tomato with TYLCV, ChiLCV and ToLCBrV^19,21^.

The begomoviruses that infect legumes in the OW, known collectively as “legume yellow mosaic viruses” (LYMVs), are amongst the most unusual of the begomoviruses (reviewed by Qazi *et al*.^24^). In phylogenetic analyses LYMVs segregate basal to all the OW begomoviruses and are distinct from the many legume-infecting begomoviruses identified in the NW^25,26^. The distinction between LYMV and other OW begomoviruses has been proposed to be due to genetic isolation; there possibly being either a virus or vector host range barrier that prevents genetic exchange between viruses that infect legumes and non-leguminous plants^24^. LYMVs are bipartite begomoviruses and the group includes *Mungbean yellow mosaic India virus* (MYMIV)^27^, *Mungbean yellow mosaic virus* (MYMV)^28^ and *Dolichos yellow mosaic virus*^29^. They occur widely across the Indian subcontinent and, in the case of MYMV, Thailand. Recently MYMV has been identified in Vietnam and MYMIV in Indonesia^30^. The LYMVs cause distinctive yellow mosaic symptoms in, and extensive losses to, grain legume production^24^.

The study detailed here has analysed, as part of the routine screening of crops for viruses, the diversity of begomoviruses associated with ToLCD affected tomato in Oman. In contrast to earlier studies, which used PCR with either universal of specific primers, the study here has used the more sensitive next generation sequencing (NGS) of circular DNAs amplified by rolling circle amplification (RCA) to identify DNA viruses in tomato. The results obtained are, broadly, consistent with earlier studies showing that the major viruses infecting tomato in the Al-Batinah region of Oman are TYLCV and ChiLCV in association with the betasatellite ToLCB. Surprisingly the analysis also identified a significant incidence of MYMIV in ToLCD affected tomato across all tomato growing regions of the country. The possible effect of the presence of MYMIV on TYLCV-ToLCB infected plants was investigated by inoculating cloned virus components to plants. The wide ranging implications of these findings are discussed.

## Results

### Preliminary screening of tomato plants for virus infection

**Figure 1:**
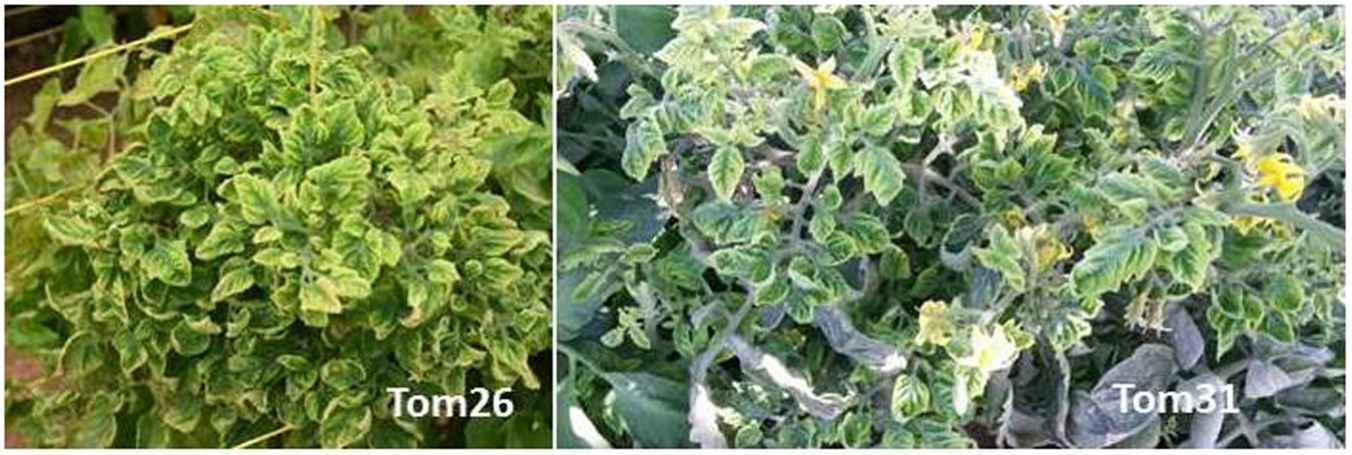
Severe tomato leaf curl disease symptoms in field-infected tomato plants (Tom26 and Tom31) from Al-Batinah, Oman.

During a survey in December 2016, fields of tomato were observed with plants showing yellowing, curling and stunting symptoms (Fig. 1) in the Barka Governorate, Al-Batinah region of Oman. Leaves from six symptomatic plants (Tom26, Tom30, Tom31, Tom32, Tom34 and Tom35), originating from four separate farms, were collected as well as leaves from a non-symptomatic plant from each farm. On the sampled farms 65–70% of tomato plants were showing typical ToLCD symptoms. Initially DNA samples extracted from leaves were screened by PCR with primer pair TYLCD-356/TYLCD-1044, designed to conserved regions of the genomes of begomoviruses prevalent in Oman. Amplification products of the expected size, ~700 nt, were obtained for PCR reactions with DNA extracted from all symptomatic samples but not from the non-symptomatic plants. This finding confirmed the association of a begomovirus with the symptoms in tomato.

### NGS of virus-positive tomato plants

DNA extracted from the six tomato samples were subjected to RCA to amplify all circular DNA molecules^31^. This yielded high-molecular weight DNA products (concatamers) for all six samples. RCA products were sequenced by DNA-seq on an Illumina HighSeq. 4000PE101platform. Raw sequencing data was processed to remove adapter sequences, low quality reads (below Phred score 20) and host-related sequences. The remaining good quality sequences were assembled *de novo*. Assembled sequences were identified using by Blast comparison to sequences available in the GenBank database. The results of the NGS (accession PRJNA531683) suggested that all the tomato plants were infected with TYLCV, ChiLCV, ToLCBrV and ToLCB (Table 1). For each sample the vast majority of the reads were mapped to TYLCV. Additionally, for at least two of the samples (Tom 26 and Tom 30), the NGS data suggested the presence of the DNA A and DNA B genomic components of MYMIV. However, in comparison to the numbers of reads which could be mapped to TYLCV, ChiLCV, ToLCBrV and ToLCB, the reads that could be mapped to MYMIV were very low (Table 1).

**Table 1:** NGS sequence reads mapped to TYLCV, ChiLCV, ToLCBrV, ToLCB and MYMIV.

Sample	Total Sequence Reads	Sequence reads mapped to -					
		TYLCV	ChiLCV	ToLCBrV	ToLCB	MYMIV DNA A	MYMIV DNA B
Tom26	46,559,532	32,337,132 (69.4%)	1,669,278 (3.6%)	9,913,951 (21.2%)	259,115 (0.55%)	1,062 (0.002%)	2,152 (0.005%)
Tom30	86,458,498	52,371,104 (60.57%)	15,529,268 (17.96%)	18,696,674 (21.62%)	268,415 (0.31%)	108 (0.00%)	1,120 (0.00%)
Tom31	83,950,036	15,854,183 (18.9%)	993,391 (1.18%)	6,370,118 (7.58%)	47,816,644 (56.9%)	4 (0.00%)	148 (0.00%)
Tom32	46,010,628	28,234,474 (61.3%)	2,680,269 (5.8%)	8,458,790 (18.4%)	218,163 (0.47%)	9 (0.00%)	15 (0.00%)
Tom34	120,459,810	88,318,623 (73.3%)	4,822,183 (4.0%)	26,595,641 (22.07%)	254,998 (0.21%)	373 (0.00%)	14 (0.00%)
Tom35	68,869,822	48,399,000 (70.27%)	5,340,667 (7.75%)	14,229,098 (20.66%)	237,902 (0.34%)	13 (0.00%)	19 (0.00%)

### Confirmation of NGS results

RCA products from the six tomato samples were digested with various restriction endonucleases. Restriction yielded ~2.7 kb DNA fragments from all samples, which were cloned in pUC19 and Sanger end sequenced. After partial sequencing and blast analysis the sequences formed three groups, representative of three distinct begomovirus species (results not shown). For each species a single clone from each tomato sample was fully sequenced using the primer-walking strategy. Analysis of these sequences is detailed in the next section.

Specific primers for the PCR-mediated amplification of MYMIV DNA A (MYMIV-AF/MYMIV-AR) and DNA B (MYMIV-BA/MYMIV-BR) were designed based on the Illumina HighSeq contigs. PCR reactions with primer pairs MYMIV-AF/MYMIV-AR and MYMIV-BA/MYMIV-BR resulted in the amplification of ~2.7 and ~2.6 kb DNA fragments, respectively, for Tom26, Tom30, Tom31 and Tom35. No amplification products were obtained for PCR reactions with DNA extracted from samples Tom32, Tom34 using primers MYMIV specific primers. This result confirmed the presence of MYMIV for the four samples and the absence of MYMIV in samples Tom32, Tom34.

### Characterization of clones of monopartite begomoviruses and betasatellite obtained from tomato with ToLCD

**Figure 2:**
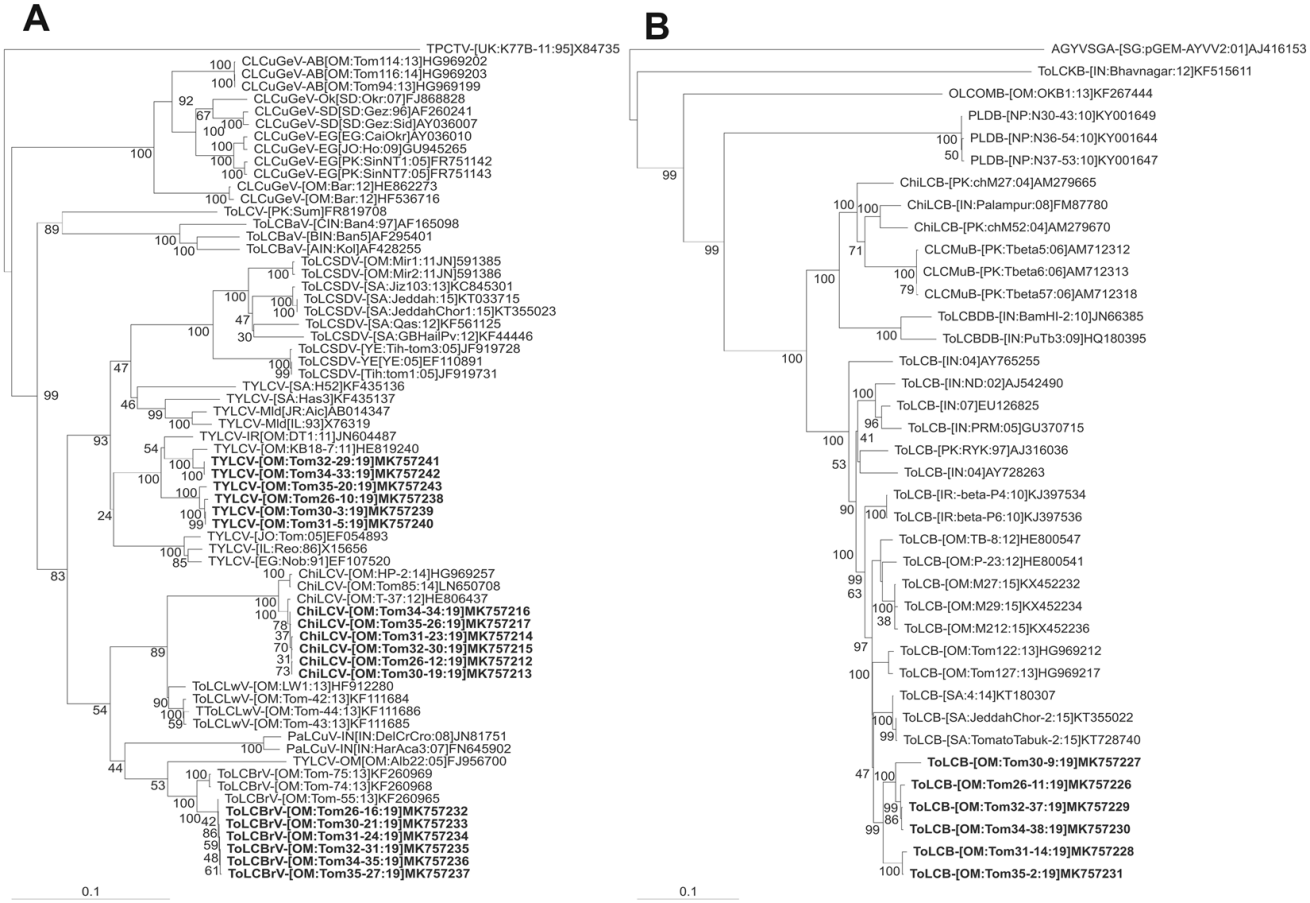
Phylogenetic dendrograms based upon alignments of the complete nucleotide sequences of the sequences of *Tomato yellow leaf culr virus* (TYLCV), *Chili leaf curl virus* (ChiLCV) and *Tomato leaf curl Barka virus* (ToLCBrV) isolated from tomato with the genome sequences of selected monopartite begomoviruses from the databases (**A**) and the sequences of *Tomato leaf curl betasatellite* (ToLCB) isolated from tomato with selected betasatellite sequences available in the databases (**B**). Begomovirus acronyms used are *Cotton leaf curl Gezira virus* (CLCuGeV), *Papaya leaf curl virus* (PaLCuV), *Tomato leaf curl Bangalore virus* (ToLCBaV), *Tomato leaf curl Liwa virus* (ToLCLwV), *Tomato leaf curl Sudan virus* (ToLCSDV) and *Tomato leaf curl virus* (ToLCV). Betasatellite acronyms used are *Chili leaf curl betasatellite* (ChiLCB), *Cotton leaf curl Multan betasatellite* (CLCuMuB), *Okra leaf curl Oman betasatellite* (OLCOMB), *Pea leaf distortion betasatellite* (PLDB), *Tomato leaf curl Bangladesh betasatellite* (ToLCBDB) and *Tomato leaf curl Karnataka betasatellite* (ToLCKB). The trees were arbitrarily rooted on the sequence of Tomato pseudo-curly top virus (TPCTV, X84735) for the virus tree and the sequence of *Ageratum yellow vein Singapore alphasatellite* (AYVSGA, AJ416153) for the betasatellite tree as outgroup. The database accession numbers are indicated in each case. The sequences originating from tomato are indicated by bold text in each case.

The sequences of six clones (Tom 26–10, Tom 30–3, Tom 31–5, Tom 32–29, Tom 34–33 and Tom 35–20), obtained from RCA products digested with *Xba*l, showed high levels of sequence identity to isolates of TYLCV available in the databases. Four sequences (Tom 30–3, Tom 31–5, Tom 26–10 and Tom 35–20), isolated from tomato plants in which MYMIV was identified, showed between 99.1 and 100% sequence identity to each other but only 94.6 to 95.4% identity to the two clones (Tom 32–29 and Tom 34–33) obtained from the tomato plants in which MYMIV was not identified. The four sequences showed the highest levels of sequence identity (99.3 to 100%) to an isolate of TYLCV strain “Iran” (TYLCV-IR) recently obtained from common bean in Oman (MG970362)^32^ whereas the two other isolates showed the highest levels of sequence identity (99.9%) to two isolates of TYLCV-IR obtained from tomato originating from Oman (DQ644565, FJ956702)^2,19^. A closer analysis of the six TYLCV-IR sequences obtained here showed the two sequences from tomato in which MYMIV was not identified to differ from the four sequences in which MYMIV was identified in the intergenic region which contain numerous sequence changes as well as several insertions and deletions (results not shown). In a phylogenetic analysis the two sequences from tomato in which MYMIV was not identified segregate apart from the other four sequences (Fig. 2A). An analysis of the six TYLCV sequences produced here from clones obtained by PCR amplification for possible recombination using the Recombination Detection Program (RDP)^33^ showed the two clones (Tom 32–29 and Tom 34–33) obtained from the tomato plants in which MYMIV was not identified to differ from the four clones from the tomato plants in which MYMIV was identified. All sequences have potential recombination events in the intergenic region and the sequences obtained from tomato plants in which MYMIV was identified additionally having a recombination event at the N-terminal end of the C4 gene (Supplementary Fig. 1). These recombination events possibly explain the differences seen between the two groups of TYLCV sequences in the phylogenetic analysis.

The sequences of six clones (Tom 26–12, Tom 30–19, Tom 31–23, Tom 32–30, Tom 34–34 and Tom 35–26) obtained from RCA product digested with *Hind*lll showed the highest levels (98.8 to 100%) of sequence identity to two ChiLCV isolates available in the databases; HG969264 isolated from radish originating from Oman^34^ and KX787939 isolated from watermelon originating from Oman^35^. In a phylogenetic analysis these sequence segregated with the sequences of ChiLCV isolates previously obtained from Oman (Fig. 2A).

Six clones (Tom 26–16, Tom 30–21, Tom 31–24, Tom 32–31, Tom 34–35 and Tom 35–27), obtained from RCA product digested with *Xba*l, showed the highest levels of sequence identity (99.7 to 100%) to an isolate of ToLCBrV from tomato in Oman^23^. In a phylogenetic analysis these sequence segregated with previously published sequences of ToLCBrV; a begomovirus species so far only identified in Oman (Fig. 2A).

Six potentially full-length clones, Tom26–11, Tom30–9, Tom31–14, Tom32–7, Tom 34–38 and Tom35–2, obtained from PCR amplifications with universal betasatellite primers^36^ were sequenced. These sequences are between 1,375 and 1,386 nt in length and are available in the GenBank database under the accession numbers given in Table 2. The six sequences show the features typical of betasatellites consisting of a single conserved open reading frame, in the complementary-sense, with the capacity to encode a product of 118 amino acids (Table 2), a region of sequence rich in adenine (coordinates 721–967) and a sequence conserved between all betasatellites, the satellite conserved region (coordinates 1255–16). All the betasatellite sequences obtained showed high nucleotide sequence identity (>90%) to ToLCB isolates previously reported from Oman. An analysis using the Species Demarcation Tool (SDT) showed the highest levels of identity to be to an isolate of ToLCB associated with *Chili leaf curl virus* from watermelon (KX787940)^35^ for Tom31–14 and Tom35–2 (99.3 and 99.6%, respectively), to an isolate associated with *Tomato leaf curl Barka virus* from tomato (KF293292)^23^ for Tom26–11 (96.6%) and to an isolate from tomato (KF229727) for Tom30–9 (94.5%). A phylogenetic analysis based upon an alignment with selected other betasatellite sequences from the databases showed the four betasatellite sequences obtained here to be most closely related to other isolates obtained from Oman, with which they form a separate clade, followed by ToLCB isolates from Iran (Fig. 2B).

**Table 2:** Features of begomovirus and betasatellite clones isolated from field infected tomato plants.

Clone	Begomovirus genome/DNA A component									DNA B/Betasatellite			
	Virus	Acc. no.^#^	Size (nt)	Position of genes [coordinates]/no. of amino acids (predicted coding capacity in kDa)									
				CP	(A)V2	Rep	TrAP	REn	(A)C4	Clone	Segment/acc. no.^#^	Size (nt)	Position of BV1/BC1 or βC1gene (coordinates)/no. of amino acids [predicted coding capacity in kDa]
Tom 26–4	MYMIV DNA A	MK757218	2,746	316–1089 257 (29.7)	141–497 118 (13.53)	1538–2626 362 (41.30)	1228–1680 150 (17.24)	1086–1490 134 (15.7)	2176–2475 99 (11.36)	Tom 26-7	MYMIV DNA B/ MK757222	2,652	419–1189 267 (30.47)/1220–2116 298 (33.76)
Tom 26–10	TYLCV	MK757238	2,767	304–1080 258 (30)	144–494 116 (13.43)	1553–2617 354 (39.2)	1222–1629 135 (16)	1077–1481 134 (15.7)	2167–2466 99 (11.36)	Tom 26–11	ToLCB MK757226	1,384	201–557 118 (13.62)
Tom 26–12	ChiLCV	MK757212	2,761	309–1082 257 (29.7)	149–514 121 (14)	1531–2616 343 (40)	1242–1628 120 (13.2)	1079–1483 127 (14.7)	2166–2459 91 (10.4)	—	—	—	—
Tom 26–16	ToLCBrV	MK757232	2,753	301–1077 258 (30)	141–491 116 (13.43)	1526–2611 361 (39.2)	1219–1623 134 (15.7)	1074–1478 134 (15.7)	2197–2454 85 (11.36)	—	—	—	—
Tom 30–6	MYMIV DNA A	MK757219	2,746	316 –1089 257 (29.7)	141–497 118 (13.53)	1538–2626 362 (41.30)	1228–1680 150 (17.24)	1086–1490 134 (15.7)	2176–2475 99 (11.36)	Tom 30–13	MYMIV DNA B/ MK757223	2,652	419–1189 267 (30.47)/1220–2116 298 (33.76)
Tom 30–3	TYLCV	MK757239	2,768	305–1081 258 (30)	145–495 116 (13.43)	1554–2618 354 (39.2)	1223–1630 135 (16)	1078–1482 134 (15.7)	2168–2467 99 (11.36)	Tom 30–9	ToLCB/ MK757227	1,376	201–557 118 (13.62)
Tom 30–19	ChiLCV	MK757213	2,761	309–1082 257 (29.7)	149–514 121 (14)	1531–2616 343 (40)	1242–1628 120 (13.2)	1079–1483 127 (14.7)	2166–2459 91 (10.4)	—	—	—	—
Tom 30–21	ToLCBrV	MK757233	2,753	301–1077 258 (30)	141–491 116 (13.43)	1526–2611 361 (39.2)	1219–1623 134 (15.7)	1074–1478 134 (15.7)	2197–2454 85 (11.36)	—	—	—	—
Tom 31–1	MYMIV DNA A	MK757220	2,746	316 –1089 257 (29.7)	141–497 118 (13.53)	1538–2626 362 (41.30)	1228–1680 150 (17.24)	1086–1490 134 (15.7)	2176–2475 99 (11.36)	Tom 31–15	MYMIV DNA B/ MK757224	2,652	419–1189 267 (30.47)/ 1220–2116 298 (33.76)
Tom 31–5	TYLCV	MK757240	2,768	305–1081 258 (30)	145–495 98 (13.42)	1554–2618 354 (39.2)	1223–1630 135 (16)	1078–1482 134 (15.7)	2168–2467 99 (11.36)	Tom 31–14	ToLCB/ MK757228	1,376	201–557 118 (13.62)
Tom 31–23	ChiLCV	MK757214	2,761	309–1082 257 (29.7)	149–514 121 (14)	1531–2616 343 (40)	1242–1628 120 (13.2)	1079–1483 127 (14.7)	2166–2459 91 (10.4)	—	—	—	—
Tom 31–24	ToLCBrV	MK757234	2,753	301–1077 258 (30)	141–491 116 (13.43)	1526–2611 361 (39.2)	1219–1623 134 (15.7)	1074–1478 134 (15.7)	2197–2454 85 (11.36)	—	—	—	—
Tom 32–29	TYLCV	MK757241	2,765	291–1067 258 (30)	131–481 116 (13.43)	1540–2604 354 (39.2)	1209–1616 135 (16)	1064–1468 134 (15.7)	2154–2447 97 (11.36)	Tom 32–37	ToLCB/ MK757229	1,376	201–557 118 (13.62)
Tom 32–30	ChiLCV	MK757215	2,761	309–1082 257 (29.7)	149–514 121 (14)	1531–2616 343 (40)	1242–1628 120 (13.2)	1079–1483 127 (14.7)	2166–2459 91 (10.4)	—	—	—	—
Tom 32–31	ToLCBrV	MK757235	2,753	301–1077 258 (30)	141–491 116 (13.43)	1526–2611 361 (39.2)	1219–1623 134 (15.7)	1074–1478 134 (15.7)	2197–2454 85 (11.36)	—	—	—	—
Tom 34–33	TYLCV	MK757242	2,765	291–1067 258 (30)	131–481 116 (13.43)	1540–2604 354 (39.2)	1209–1616 135 (16)	1064–1468 134 (15.7)	2154–2447 97 (11.36)	Tom 34–38	ToLCB/ MK757230	1,376	201–557 118 (13.62)
Tom 34–34	ChiLCV	MK757216	2,761	309–1082 257 (29.7)	149–514 121 (14)	1531–2616 343 (40)	1242–1628 120 (13.2)	1079–1483 127 (14.7)	2166–2459 91 (10.4)	—	—	—	—
Tom 34–35	ToLCBrV	MK757236	2,753	301–1077 258 (30)	141–491 116 (13.43)	1526–2611 361 (39.2)	1219–1623 134 (15.7)	1074–1478 134 (15.7)	2197–2454 85 (11.36)	—	—	—	—
Tom 35–8	MYMIV DNA A	MK757221	2,748	316–1089 257 (29.7)	141–497 118 (13.53)	1539–2627 362 (41.30)	1229–1681 150 (17.24)	1108–1491 127 (14)	2177–2476 99 (11.36)	Tom 35–18	MYMIV DNA B/ MK757225	2,652	419–1189 267 (30.47)/1220–2116 298 (33.76)
Tom 35–20	TYLCV	MK757243	2,755	292–1068 258 (30)	132–482 116 (13.43)	1541–2605 354 (39.2)	1210–1617 135 (16)	1065–1469 134 (15.7)	2155–2430 99 (11.36)	Tom 35–2	ToLCB/ MK757231	1,375	201–557 118 (13.62)
Tom 35–26	ChiLCV	MK757217	2,761	309–1082 257 (29.7)	149–514 121 (14)	1531–2616 343 (40)	1242–1628 120 (13.2)	1079–1483 127 (14.7)	2166–2459 91 (10.4)	—	—	—	—
Tom 35–27	ToLCBrV	MK757237	2,753	301–1077 258 (30)	141–491 116 (13.43)	1526–2611 361 (39.2)	1219–1623 134 (15.7)	1074–1478 134 (15.7)	2197–2454 85 (11.36)	—	—	—	—

^#^GenBank database accession number.

Efforts to identify the presence of possible alphasatellites, either by PCR with universal primers for alphsatellites^37^ or by cloning from RCA products, were uniformly negative.

### Characterization of MYMIV clones obtained from tomato with ToLCD

The PCR amplicons obtained with primers specific for MYMIV DNA A and DNA B were cloned into pTZ57R/T. Eight potentially full-length clones, four (Tom26–4, Tom30–6, Tom31–1 and Tom35–8) obtained with the DNA A primers and four (Tom26–7, Tom30–13, Tom31–15 and Tom35–18) obtained with the DNA B primers, were selected for further analysis. The eight clones were sequenced in their entirety and the sequences are available in the GenBank sequence database under the accession numbers given in Table 2.

**Figure 3:**
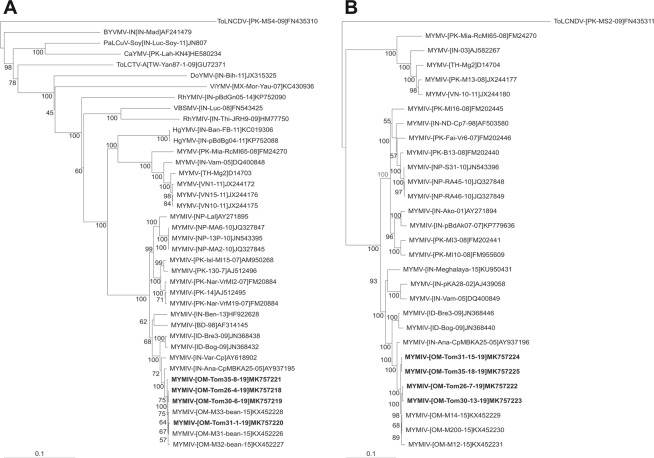
Phylogenetic dendrograms based upon alignments of the complete nucleotide sequences of the DNA A sequences of *Mungbean yellow mosaic India virus* (MYMIV) isolated from tomato with the genome or DNA A sequences of selected begomoviruses from the databases (**A**) and the DNA B sequences of MYMIV isolated from tomato with selected DNA B components of begomoviruses available in the databases (**B**). Vertical branches are arbitrary; horizontal branches are proportional to calculated mutation distance. Values at nodes indicate percentage boot strap values (1000 replicates). Begomovirus acronyms used are *Bhendi yellow vein mosaic virus* (BYVMV), *Catharanthus yellow mosaic virus* (CaYMV), *Dolichos yellow mosaic virus* (DoYMV), *Horsegram yellow mosaic virus* (HgYMV), *Mungbean yellow mosaic virus* (MYMV), *Papaya leaf curl virus* (PaLCuV), *Rhynchosia yellow mosaic India virus* (RhYMIV), *Rhynchosia yellow mosaic virus* (RhYMV), *Tomato leaf curl Taiwan virus* (ToLCTV), *Velvet bean severe mosaic virus* (VBSMV) and *Vigna yellow mosaic virus* (ViYMV). The trees were arbitrarily rooted on the DNA A sequence of *Tomato leaf curl New Delhi virus* (ToLCNDV-[PK:MS4:09]FN435310) for the DNA A tree and DNA B of ToLCNDV-[PK:MS2:09]FN435311for the DNA B tree as outgroup. The database accession numbers are indicated in each case. The sequences originating from tomato are indicated by bold text in each case.

The sequences of clones Tom26–4, Tom30–6, Tom31–1 are 2,746 nt in length and that of Tom35–8 is 2,748 nt. The sequences have all characteristics typical of the genomes/DNA A components of begomoviruses originating from the Old World, encoding four genes in the complementary-sense and two in the virion-sense orientation (Table 2). The four sequences showed greater than 99.5% nucleotide identity with each other while and the highest nucleotide sequence identities (99 to 99.3% %) with the DNA A components of MYMIV isolates previously identified in Oman^38,39^, followed by 99 to 99.3% identity to the DNA A sequence of a MYMIV isolate from cowpea in India (AY937195)^40^. Based on the presently applicable criteria for species demarcation of begomoviruses Tom26–4, Tom30–6, Tom31–1 and Tom35–8 are the DNA A component of isolates of MYMIV^41^. In a phylogenetic analysis the four sequences from tomato segregated with the DNA A components of other MYMIV isolates available in the databases, with the closest relationship, and forming a discrete clade, with isolates previously characterized from Oman, isolates originating from Indonesia, three isolates from India and an isolate from Bangladesh (Fig. 3A).

The sequences of the four clones obtained with MYMIV DNA B specific primers (Tom26–7, Tom30–13, Tom31–15 and Tom35–18) were each of 2,652 nt in length and are available in the GenBank database under the accession numbers given in Table 2. Analysis of the four sequences with ORF Finder showed them to have an arrangement typical of the DNA B components of bipartite begomoviruses, encoding one gene in each orientation (Table 2) and to share greater than 99.2–99.8% nucleotide sequence identity. An SDT analysis of selected MYMIV and MYMV DNA B sequences available in the nucleotide sequence databases showed the DNA B sequences obtained from Oman to have the highest nucleotide sequence identities (98.8%) with the DNA B sequences of MYMIV isolates previously reported in Oman^38,39^ followed by 98–98.5% to a MYMIV DNA B sequence isolated from cowpea in India (AY937196)^40^. A phylogenetic analysis of the DNA B sequences showed the isolates from tomato to be most closely related to isolates previously identified in Oman. All the isolates from Oman were most closely related to AY937196^40^ and formed a distinct clade with the DNA B sequences of MYMIV isolates from Indonesia, some isolates from India as well as some isolates of MYMV (Fig. 3B).

The MYMIV sequences obtained by NGS agreed well with the sequences obtained from Sanger sequencing of clones. For example, for plant Tom 26 the DNA A determined by NGS showed 99.7% identity to Tom26–4 whereas the DNA B sequence showed 99.2% identity to Tom26–7. The sequences of the MYMIV DNA A and DNA B determined by NGS are given in Supplementary Fig. 2. The sequence differences likely are due to natural sequence variation in the populations of viral molecules in the plant.

### Geographical incidence of MYMIV in tomato in Oman

To determine the geographical incidence of infection of tomato with MYMIV a total of 82 tomato plants (collected between 2015 and 2016), originating from across Oman, were screened by PCR with primers to detect MYMIV. A total of 21 tomato plants screened showed the presence of either MYMIV DNA A and/or MYMIV DNA B, giving an incidence of 25% (Supplementary Table 1). The samples were collected from across the country, including the main agricultural area in Al Batinah Governorate and in the south around Salalah (Dhofar province), showing the presence of MYMIV in tomato to be widespread.

### Analysis of the infectivity of the MYMIV, TYLCV and ToLCB clones isolated from tomato

**Figure 4:**
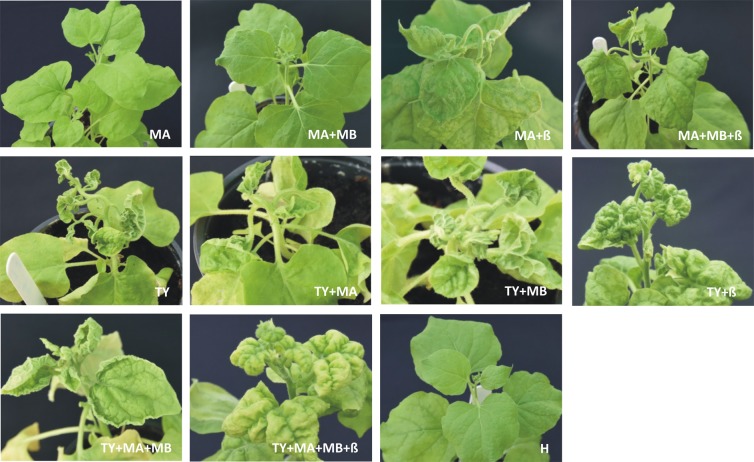
Symptoms induced in *N. benthamiana* plants following the *Agrobacterium*-mediated inoculation with constructs for the infectivity of MYMIV, TYLCV and ToLCB. Plants were either not inoculated (H) or inoculated with constructs for the infectivity of MYMIV DNA A (MA), MYMIV DNA B (MB), ToLCB (β) or TYLCV (TY) as indicated on each photograph. Photographs were taken at 24 dpi.

**Figure 5:**
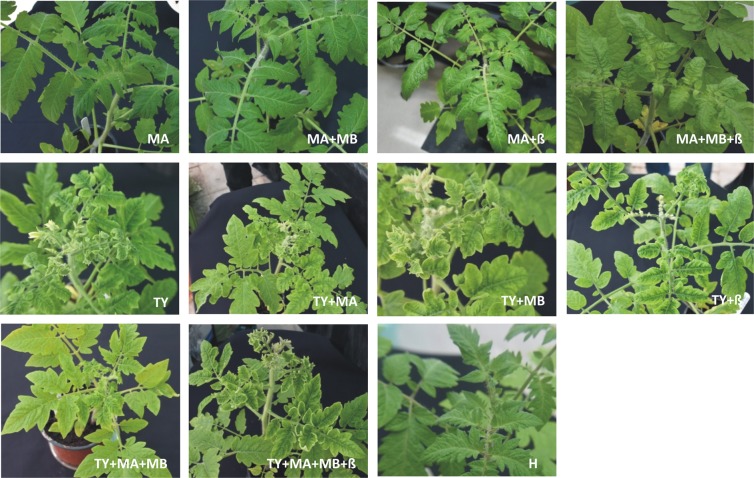
Symptoms induced in tomato plants following the *Agrobacterium*-mediated inoculation with constructs for the infectivity of MYMIV, TYLCV and ToLCB. Plants were either not inoculated (H) or inoculated with constructs for the infectivity of MYMIV DNA A (MA), MYMIV DNA B (MB), ToLCB (β) or TYLCV (TY) as indicated on each photograph. Photographs were taken at 32 dpi.

*Agrobacterium*-mediated inoculation of just the DNA A component of the MYMIV isolate from tomato to *N. benthamiana* plants resulted no apparent symptoms of infection at 24 days post inoculation (dpi) (Fig. 4; Table 3). However, for a single plant, out of seventeen inoculated plants, the presence of the DNA A component was shown by PCR diagnostics. Similarly there were no apparent symptoms following inoculation with both the DNA A and DNA B components of MYMIV, although more plants (n = 3) were found to harbour the DNA A at 24 dpi (Fig. 5; Table 3). Of these only two plants were also found to harbour the DNA B component.

**Table 3:** Infectivity of MYMIV, TYLCV and ToLCB in agroinoculated *N. benthamiana* and tomato plants.

Inoculum	*N. benthamiana*			Tomato		
	Plants symptomatic/(plants infected)^#^/plants inoculated	Symptoms*	Latent period^$^ (days)	Plants symptomatic/(plants infected)^#^/plants inoculated	Symptoms*	Latent period^$^ (days)
MYMIV A	0/(1)/17	Ns	—	0/(0^+^)/18	Ns	—
MYMIV A + MYMIV B	0/(3, 2)/18	Ns	—	0/(0^+^, 0^+^)/15	Ns	—
MYMIV A + ToLCB	10/(10, 10)/18	mCr, mDlc, Vt	16	2/(2^+^, 2)/18	mDlc, Y	28
MYMIV A + MYMIV B + ToLCB	16/(16, 4, 16)/18	mCr, mDlc, Vt	15	4/(4^+^, 2^+^, 4)/16	mDlc, Y	28
TYLCV	15/(15)/15	sUlr, Y	9	18/(18)/18	sDlc, Y	12
TYLCV + MYMIV A	18/(18, 0)/18	sUlr, Y	10	16/(16, 0^+^)/16	mUlr, Y	16
TYLCV + MYMIV B	18/(18, 0)/18	sUlr, Y	10	18/(18, 0^+^)/18	mUlr, Y	18
TYLCV + ToLCB	16/(16, 15)/16	sCr, sDlc,Vt, Y	8	15/(15, 14)/15	Dlc, Y	10
TYLCV + MYMIV A + MYMIV B	17/(17, 3, 1)/18	sUlr, Y	12	16/(16, 2^+^, 2^+^)/16	mUlr, Y	14
TYLCV + MYMIV A + MYMIV B + ToLCB	18/(18, 5, 2, 17)/18	sCr, sDlc, Vt, Y	10	17/(17, 4^+^, 2^+^, 16)/18	Dlc, Y	12

^#^Infected plants detected by diagnostic PCR. The results are from 3 independent experiments. In each case the number of plants in which each virus/virus component/betasatellite was detected is given. The virus/virus component/betasatellite are given as TYLCV (TY), MYMIV DNA A (MA), MYMIV DNA B (MB) and ToLCB (β).

^+^Number of plants positive for the MYMIV component determined by RCA-PCR following a negative result by PCR.

^*^Symptoms are denoted as foliar crumpling (Cr), downward leaf curling (Dlc), upward leaf rolling (Ulr), vein thickening (Vt), yellowing (Y) and no symptom (Ns). These terms may be described as either mild (m) or severe (s). Note that the symptoms described are for the plants harbouring the virus/virus components/betasatellite with which the plants were inoculated; in some plants one or more of the inoculated virus/virus components/betasatellite was not maintained.

^$^Time between inoculation and first appearance of symptoms.

Agroinoculation of MYMIV DNA A and ToLCB to *N. benthamiana* led to the majority of plants (10 plants out of 18 inoculated) showing symptoms of infection at 16 dpi, consisting of mild foliar crumpling, downward leaf curling and vein thickening (Fig. 5; Table 3). Diagnostic PCR further confirmed the association of MYMIV DNA A component and ToLCB with the symptomatic *N. benthamiana* plants. Co-inoculation of *N. benthamiana* with MYMIV DNA A, DNA B and ToLCB induced symptoms that were qualitatively similar to those induced without the DNA B in 16 out of 18 inoculated plants (Fig. 5). However, the symptoms were slightly milder, with less pronounced upward leaf curling, and appeared one day earlier (Table 3). Although diagnostic PCR showed the DNA A component to be present in all (n = 16) symptomatic plants the DNA B was only detected in four plants and the betasatellite in 12 plants.

All *N. benthamiana* plants (n = 15) inoculated with a TYLCV isolate from tomato obtained in this study developed symptoms of infection, consisting of a reduction in leaf size, severe upward leaf curling of leaf margins and yellowing, at 9 dpi. Inoculation of *N. benthamiana* plants with TYLCV and ToLCB induced severe downward leaf curling, leaf crumpling and yellowing in all inoculated plants (n = 16) at 8 dpi. Diagnostic PCR showed the presence of TYLCV DNA in all plants but ToLCB was only detected in 15 plants (Fig. 5; Table 3). For plants inoculated with TYLCV and either the DNA A or DNA B of MYMIV, the symptoms of infection were indistinguishable from plants inoculated with only TYLCV and in none of the plants were the MYMIV components detected by diagnostic PCR. However, the latent period was possibly one day longer than for plants inoculated with only TYLCV (10 days in comparison to 9 days, respectively). Co-inoculation of TYLCV with both the DNA A and DNA B components of MYMIV induced symptoms that were qualitatively similar to *N. benthamiana* plants inoculated with only TYLCV. However, the symptoms were a little less severe and were delayed in comparison to plant inoculated with only TYLCV (12 days compared to 9 days, respectively; Fig. 5; Table 3). When ToLCB was coinoculated with TYLCV and MYMIV DNA A and DNA B, *N. benthamiana* plants developed symptoms indistinguishable from the symptoms induced by a TYLCV with ToLCB infection, although the symptoms were delayed by approx. 2 days (Fig. 5; Table 3).

Agroinoculation of tomato plants with either DNA A alone or with MYMIV DNA A and DNA B did not lead to symptoms and neither DNA A nor DNA B were detected by diagnostic PCR or by PCR reactions using RCA products, obtained from amplifications with DNA extracted from inoculated plants, as template (RCA-PCR; Fig. 5; Table 3). In contrast, agroinoculation of tomato with either MYMIV DNA A and ToLCB or with MYMIV DNA A, DNA B and ToLCB led to very late developing symptoms (approx. 28 dpi) consisting of mild downward leaf curling and mild yellowing for a few plants (Fig. 5; Table 3). Neither of the virus components nor the betasatellite were detected in diagnostic PCR. However RCA-PCR showed the presence of the virus components in the plants with symptoms. The TYLCV isolate obtained here was highly infectious to tomato and induced severe downward leaf curling and yellowing symptoms by 12 dpi in all inoculated plants. Co-inoculation of TYLCV with ToLCB reduced the latent period (10 days) and altered the symptoms to mild downward leaf curling with slightly more yellowing. Maintenance of the betasatellite by TYLCV was shown in 14 out of 16 tomato plants inoculated by diagnostic PCR (Fig. 5; Table 3). Co inoculation of tomato plants with TYLCV and either MYMIV DNA A or MYMIV DNA B resulted in mild downward leaf curling and yellowing symptoms and extended the latent period over plants inoculated with only TYLCV, with the greatest effect seen for MYMIV DNA B (18 days). However, only TYLCV was detected in inoculated plants by diagnostic PCR and RCA-PCR. Similarly, inoculation of tomato plants with TYLCV, MYMIV DNA A and DNA B resulted in mild downward leaf curling and yellowing symptoms and extended the latent period (14 days) but both MYMIV DNA A and DNA B were detected in only two plants by RCA-PCR. Inoculation of tomato plants with all four components (TYLCV, MYMIV DNA A, MYMIV DNA B and ToLCB) induced symptoms that were qualitatively equivalent to plants inoculated with TYLCV with ToLCB but slightly milder and with a slightly longer latent period (12 days) than in the absence of the betasatellite. Again MYMIV DNA A (in four plants) and MYMIV DNA B (in two plants) were detected in these plants although both TYLCV and ToLCB were detected in the majority of plants.

## Discussion

During a small survey of tomato crops conducted during 2015 in the Al-Batinah region of Oman using NGS, the presence in some plants of monopartite begomoviruses (TYLCV, ChiLCV, ToLCBrV), a betasatellite (ToLCB) and a bipartite begomovirus (MYMIV) was shown. The NGS results were confirmed by cloning the viruses/virus components and betasatellite, either from RCA product or by PCR-mediated amplification. TYLCV (specifically the “Iran” and “Oman” strains), ChiLCV and ToLCBrV are common monopartite begomoviruses infecting tomato, causing ToLCD, in Oman and are frequently associated with ToLCB, the only betasatellite so far shown to infect tomato in Oman^19,20^. The finding of these viruses and betasatellite in tomato is thus not surprising. However, the identification of co-infection of these viruses and betasatellite with MYMIV in tomato is surprising.

The incidence of ToLCD in the survey fields in Al-Batinah during 2015 was between 65 and 70% and of the six tomato plants examined by NGS four were found to harbour MYMIV. A more extensive survey for MYMIV in tomato, for samples collected in 2015 and 2016, indicated that approx. 25% of symptomatic plants harboured MYMIV and showed that the phenomenon was occurring not only in the major agricultural area of Al-Batinah (north Oman) but also in the agricultural area in southern Oman (Dhofar region).

In the phylogenetic analyses the MYMIV DNA A and DNA B components originating from Oman, including those identified earlier^38,39^, form a distinct clade with MYMIV isolates from Indonesia and some isolates from India and Bangladesh. All these isolates are unusual in that, as first noted by John *et al*.^40^ and later confirmed for the isolates from Indonesia by Tsai *et al*.^30^, they are MYMIV but with a DNA B component derived from the closely related MYMV, likely by a process known as pseudo-recombination (component exchange)^26^. This was confirmed for the Oman isolates by alignments, which show the majority of the DNA B sequence to have the highest levels of identity to the sequences of MYMV DNA B components but the intergenic region to have higher levels of sequence identity to MYMIV isolates (results not shown).

Based on their results Tsai *et al*.^30^ concluded that MYMIV was introduced into Indonesia only very recently. The collective results from Oman similarly suggest that MYMIV has only been introduced quite recently. The very close relationship of the isolates in Oman and Indonesia also suggests that they have a common origin, likely having been introduced from South Asia. The likely mechanism of introduction is unclear, although the demonstration for an increasing number of geminiviruses that seed transmission can occur, including for the LYMVs DoYMV^42^ and possibly MYMV^43^, which are closely related to MYMIV, may suggest that the mechanism of geographic dispersal was via seed.

In Oman both TYLCV-IR and MYMIV have previously been identified separately in legumes; common bean and kidney bean, respectively^32,39^. Although generally considered a tomato virus, TYLCV can infect and causes problems in legumes; for example the type strain of TYLCV is one of two viruses shown to cause bean leaf crumple disease in Spain^44^. The unusual aspect of the findings of Shahid *et al*.^39^ was the identification of MYMIV with a betasatellite (ToLCB) in kidney bean. This virus is rarely encountered in the presence of a betasatellite. Rouhibakhsh and Malathi^45^ showed MYMIV in cowpea with *Cotton leaf curl Multan betasatellite* (CLCuMuB) and Ilyas *et al*.^46^ showed the virus in soybean with *Tobacco leaf curl betasatellite* (TobLCB). In all three cases the symptoms of the infections suggested that the betasatellite was enhancing symptoms –the symptoms in each case having features not usually associated with MYMIV infection. MYMIV has on one occasion been identified in tomato without, apparently, the presence of a betasatellite^47^. The MYMIV identified in tomato in that case was also of the pseudo-recombinant type, with a DNA B originating from MYMV.

The infectivity analysis with cloned virus/virus components/betasatellite shows that both *N. benthamiana* and tomato are non-hosts of MYMIV. For *N. benthamiana*, both components could be detected in a small number of plants after inoculation with the DNA A and DNA B components of MYMIV, although no symptoms were induced. The inability of MYMIV, and other LYMVs, to infect *N. benthamiana* has been demonstrated previously^29,48,49^. However, in the presence of ToLCB MYMIV DNA A spread efficiently in *N. benthamiana* plants and induced symptoms. When MYMIV DNA B was included in the inoculum with DNA A and ToLCB, the DNA B was maintained in a few plants. This suggests that the inability of MYMIV to infect *N. benthamiana* is due to a deficiency in DNA B. Since the DNA B component of bipartite begomoviruses encodes proteins involved in virus movement in plants^11^, the deficiency of MYMIV may be in the ability to spread. Certainly the efficient spread (infectivity) of the DNA A component in the presence of the betasatellite supports this. Betasatellites have previously been shown to complement DNA B functions suggesting that the only protein encoded by betasatellites, βC1, has movement functions^50–52^. However, the demonstration that βC1has suppressor of both post-transcriptional and transcriptional gene silencing^17^ may indicate that rather than providing movement functions, betasatellites instead overcome a host resistance to movement based on gene silencing. This is supported by the finding here that inoculation with the betasatellite reduces the latent period (faster spread of the virus following inoculation) and alters the phenotype of TYLCV infection (indicating differing plant tissues affected and/or timing of the infection of those tissues), as noted previously for a number of monopartite begomovirus-betasatellite interactions including TYLCV/ToLCB^2,53–55^.

In co-inoculations with TYLCV, MYMIV was only maintained in *N. benthamiana* in the presence of ToLCB or in inoculations that also included MYMIV DNA B. However, in inoculations with MYMIV DNA B, the DNA B was not maintained in most plants. This suggests that, although not well adapted to *N. benthamiana*, this component does contribute to the infectivity of the DNA A (more plants that ultimately contain the DNA A).

For inoculations of tomato MYMIV DNA A and/or DNA B were only maintained in the presence of ToLCB. In the few plants that MYMIV DNA A and DNA B were detected the genomic components were below the detection threshold of PCR, indicating that the titres were very low. Nevertheless, in the inoculations with MYMIV DNA A and/or DNA B there were changes in the latent periods and symptoms exhibited by infections of TYLCV and TYLCV/ToLCB which may suggest that the MYMIV components are affecting the infection. However, this aspect will require further investigation.

The earlier identification of both TYLCV-IR/ToLCB^32^ and MYMIV/ToLCB^39^ in *Phaseolus vulgaris* (kidney bean and common bean, respectively) in the same geographic area provides a likely route (insect transmission from legumes) for the co-infection of tomato occurring. This is supported by the finding here that the TYLCV-IR isolates from tomato in which MYMIV was not identified show closer relatedness to TYLCV-IR isolates earlier identified in tomato, whereas TYLCV-IR isolates from tomato plants in which MYMIV was identified show closer relatedness to TYLCV-IR isolates very recently identified in a legume. Whether this indicates that maintenance of MYMIV in tomato relies on a specific variant of TYLCV will require further investigation.

The host range of MYMIV does not extend to tomato^24^. In co-infected plants it is likely that proteins which suppress host defenses, including gene silencing (also known as RNA interference), encoded by TYLCV and/or the betasatellite allow MYMIV to infect tomato; a phenomenon known as *trans*-complementation^17,56–60^. However, the likely effects and stability of the TYLCV/ToLCB-MYMIV interaction are less clear. Previous studies of the interaction of bipartite begomoviruses with betasatellites has suggested that these might not be stable, either because of a conflict between the betasatellite and the DNA B^51^ or due to a conflict between the virus encoded coat protein and the betasatellite^52^. Whether the TYLCV/ToLCB-MYMIV co-infection will prove to be stable in the field is unclear, although the low levels of MYMIV components detected in tomato in the field and by agroinoculation would suggest not.

The findings here with MYMIV in tomato in Oman very much mirrors the situation with *Tomato leaf curl New Delhi virus* (ToLCNDV) in cotton in Pakistan reported recently^61^. Cotton leaf curl disease in South Asia is caused by various monopartite begomoviruses with a specific betasatellite, *Cotton leaf curl Multan betasatellite* (CLCuMuB; reviewed by Sattar *et al*.^62^). Zaidi *et al*.^61^ showed that recently, at high incidence and occurring across a wide area, CLCuD affected cotton also harbours the bipartite begomovirus ToLCNDV. The study additionally showed that, in co-infected cotton, the levels of CLCuMuB were raised. Since the betasatellite encodes a dominant symptom determinant, enhanced betsatellite titres could result in greater losses to cotton production. Whether the enhanced levels of betasatellite are also occurring in the TYLCV/ToLCB-MYMIV interaction will require further investigation.

Although the potential losses to legume and tomato production from MYMIV infection in Oman are clearly of concern, the results presented here have more far reaching implications. TYLCV has been spread around the world due to the spread of the vector whitefly, the international trade in tomato seedlings and possibly even tomato fruit^4,63,64^. The fear that a disease enhancing, TYLCV-adapted betasatellite such as ToLCB could spread with the virus, has been noted previously^18,65^. The results here raise the additional possibility that other viruses, including viruses not adapted to plants of the *Solanaceae* such as MYMIV, could be moved around the world as a passenger to TYLCV in tomato. In that regard it is pertinent to mention *Watermelon chlorotic stunt virus* (WmCSV); a bipartite OW begomovirus that has recently been shown to be present, and causing problems, in Mexico^66^. WmCSV occurs across the Arabian Peninsula, North Africa and the Middle East and has been identified co-infecting tomato with *Squash leaf curl virus*, a bipartite begomovirus originating from the NW, in Jordan^67^. The study, however, did not show the presence of TYLCV. Some evidence was obtained in the study here to indicate that one tomato plant (Tom31) was infected with WmCSV – partial sequences of the DNA A identified in the NGS (Supplementary Fig. 3), although this was not confirmed by cloning. The mechanism of introduction of WmCSV into the NW is unclear and it is intriguing to speculate that it might have been by co-infection with TYLCV in tomato.

An important question that needs to be answered with respect to tomato acting as a “vector” for the spread of viruses other than TYLCV is whether coinfected plants can act as a source of, in this case, MYMIV for *B. tabaci* insects to transmit to other plant species when the “passenger” virus is apparently at such low titre. This will be the focus of future studies.

## Materials and Methods

### Detection of viruses and betasatellite

During a survey for begomoviruses in January 2015, tomato plants showing typical ToLCD infections were collected from the Barka Governorate, Al-Batinah region of Oman. Total nucleic acid was isolated from tomato leaf samples using a CTAB-based method^68^. DNA extracts were resuspended in sterile distilled water and stored at −20 °C. The nucleic extracts were used as a template in polymerase chain reaction (PCR) in initial screening with primer pair TYLCD-356/TYLCD-1044^69^, designed to conserved sequences of tomato-infecting begomoviruses in Oman.

### Next-generation sequencing

Circular DNA molecules in nucleic acid samples were amplified by rolling circle amplification (RCA) using a TempliPhi 100 Amplification System kit (GE Healthcare) as described by the manufacturer. Resulting high molecular weight products were sent to the Beijing Genomics Institute (Hong Kong) for sample preparation and paired-end 101 sequencing on an Illumina HighSeq. 4000 platform. NGS data received in fastq format was processed by using Trimgalore (https://www.bioinformatics.babraham.ac.uk/projects/trim_galore/) on a High Performance Computing cluster and adapter sequences and low quality (Phred score <20) sequence reads were removed. Sequence reads were mapped to the tomato genome sequence (*Solanum lycopersicum* iTAG2.4) using Bowtie2^70^ to remove host related sequences and enrich viral sequences. Sequence reads not mapping to the tomato genome sequence were isolated and *de-novo* assembled using SPAdes version 3.12.0^71^.Contigs obtained from this assembly were identified by using stand-alone Blast search tool.

### Amplification by PCR, RCA and cloning of viruses and betasatellite

MYMIV infection of tomato plants was detected by PCR with primers for the detection of MYMIV DNA A (MYMIV-CPF [5′-TCCCCCGGGATGCCAAAGCGGACCTAC-3′]/MYMIV-CPR [5′-CAAGTCGACTAATTCAATATCGAATCA-3′]) and MYMIV DNA B (MYMIV-NSPF [5′-AACATCGATATGAAGGCCATGAACGTGA-3′]/MYMIV-NSPR [5′-TTAGTCGACTTATCCAACGTATTTCA-3′]). The full length components of MYMIV were PCR-amplified from DNA extracts using specific primes MYMIV-AF (5′-GTAAAGCTTACATCCTCCACCAGGTG-3′)/MYMIV-AR (5′-TGTAAGCTTTACGCATAATGTTCAATAC-3′) for the DNA A component and MYMIV-BA (5′-CAGGATCCAATGATGCCTCTGGCA-3′)/MYMIV-BR (5′-TTGGATCCTGGAGATTCAATATCT-3′) for the DNA B component. These primers for the amplification of full-length components were designed to the sequences obtained from the NGS analysis. Betasatellites were amplified with primer pair beta01/beta02^36^. The amplification products were cloned in pTZ257R/T (Fermentas). Concatameric RCA products were digested with restriction endonucleases *Xba*I or *Pst*I. Resulting ~2.7 kb fragments were purified from agarose gels using a GeneJet Gel Extraction Kit (Thermo Fisher Scientific) and cloned in pUC19^72^.

### Sanger sequencing, sequence assembly and sequence analysis

Selected clones harbouring potentially full-length begomovirus or satellite inserts were sequenced commercially (Macrogen Inc., South Korea). Sequence reads were assembled using SeqMan, part of the Lasergene package of sequence analysis software (DNA Star Inc., Madison, WI, USA). The resultant sequences were initially analyzed using BLASTn (http://blast.ncbi.nlm.nih.gov/Blast.cgi)^73^ to identify closely related sequences in the nucleotide sequence databases. Percentage sequence identity values quoted were determined using SDT with the MUSCLE alignment option^74,75^. Pairwise multiple sequence alignments using the MUSCLE algorithm were produced using MEGA6^76^. Evolutionary relationships were determined by constructing phylogenetic trees using Clustal X (neighbor-joining method^77^) and displayed using Treeview^78^. Possible recombination events in sequences were determined using RDP4^33^. Open reading frames (ORFs) in sequences were identified using ORF Finder (https://www.ncbi.nlm.nih.gov/orffinder).

### Production of constructs for Agrobacterium-mediated inoculation

A full-length clone of MYMIV DNA A (Tom26–4) was digested with *Hin*dIII and *Xho*I to release a fragment of ~1,600 bp that was ligated to into the binary vector pGreen0029^79^ to yield pGMYA-0.4. Then the full-length insert of Tom26–4 was released using *Hin*dIII and ligated into pGMYA-0.4, linearised with *Xho*I, to yield the partial direct repeat construct pGMYA-1.4. Similarly, MYMIV DNA B (Tom26–7) was digested with *Bam*HI and *Cla*I to release a fragment of ~1, 700 bp and ligated to into pGreen0029 to yield pGMYB-0.4 into which the full-length insert of Tom26–7, released with *Bam*HI, was ligated to yield pGMYB-1.4. A partial direct repeat construct of TYLCV (Tom26–10) in pGreen0029 was produced using an ~1.249 bp *Bam*HI - *Xba*I fragment and the full-length insert of Tom26–10, released using *Xba*I, to yield pGTY-1.4. A partial direct repeat construct of ToLCB (Tom26–11) in pGreen0029 was produced using an ~600 bp *Bam*HI-*Xba*I fragment and the full-length insert released using *Kpn*I to yield pGTB-1.4. The pGreen0029 constructs were finally electroporated into *Agrobacterium tumefaciens* strain LBA4444.

### Agrobacterium-mediated inoculation and maintenance of plants

*Agrobacterium* cultures harbouring the pGreen0029 constructs were grown for 48 h in 50 ml LB liquid medium supplemented with which antibiotics (kanamycin 25 µg/ul, tetracycline 10 µg/ul, rifampicin 50 µg/ul). The bacteria were pelleted by centrifugation at 4800 g for 10 min at 4 °C and resuspended in infiltration solution (10 mM MgCl_2_, 200 µM acetosyringone) and adjusted to a final optical density at 600 nm of ~1.0. Agroinoculation was performed on 4–5 week old *N. benthamiana* and tomato (*Solanum lycopersicum* L. cv. Pusa Ruby) plants as described previously^2^. For each combination of begomovirus and/or betasatellite, 5–6 plants were used for inoculation. Inoculated plants were maintained in a secure, insect-free growth room with a day length of 18 h, a day/night temperature of 25/26 °C and a relative humidity of 65%. The plants were observed daily for the appearance of symptoms of virus infection.

## Supplementary information

Supplementary Table 1

Supplementary Figures 1–3
