# Supplementary material for: Frequent occurrence of Mungbean yellow mosaic India virus in tomato leaf curl disease affected tomato in Oman
Source: Sci Rep. 2019 Nov 12;9:16634. doi: 10.1038/s41598-019-53106-4 (PMC6851148; doi:10.1038/s41598-019-53106-4)
Supplement: Supplementary file 1 — Supplementary Table 1 [file 41598_2019_53106_MOESM1_ESM.docx]

**Frequent occurrence of *Mungbean yellow mosaic India virus* in tomato leaf curl disease affected tomato in Oman**

M.S. Shahid^1*#^, M. Shafiq^1^, M. Ilyas^2^, A. Raza^1^, M. N. Al-Sadrani^1^, A.M. Al-Sadi^1^, and R.W. Briddon^3#^

^1^Department of Crop Sciences, College of Agricultural and Marine Sciences, Sultan Qaboos University, Al-Khod 123, Oman.

^2^Cell Biology and Molecular Genetics, University of Maryland College Park, MD 20742 USA

^3^Agricultural Biotechnology Division, National Institute for Biotechnology and Genetic Engineering, Faisalabad, Pakistan.

**Supplementary Table 1** Sample location, sample codes, geographical co-ordinates and distribution of begomoviruses and associated tomato leaf curl betasatellite

| Sample location | Sample code | Geographical  coordinates | MYMIV  DNA A/DNA B | TYLCV | ToLCB | ChiLCV | ToLCBrV |
| --- | --- | --- | --- | --- | --- | --- | --- |
| Al-Swayq | Tom1 | 23.82^o^ N, 57.43^o^E | - | - | - | - | + |
| Al-Swayq | Tom2 | 23.82^o^ N, 57.43^o^E | +/- | - | + | + | - |
| Al-Batinah | Tom3 | 24.34^o^ N, 56.72^o^E | - | + | - | + | - |
| Al-Batinah | Tom4 | 24.34^o^ N, 56.72^o^E | - | + | + | - | - |
| Al-Batinah | Tom5 | 24.34^o^ N, 56.72^o^E | - | - | - | + | - |
| Al-Batinah | Tom6 | 24.34^o^ N, 56.72^o^E | - | - | - | - | + |
| Al-Batinah | Tom7 | 24.34^o^ N, 56.72^o^E | - | - | + | + | - |
| Al-Batinah | Tom8 | 24.34^o^ N, 56.72^o^E | - | - | + | + | - |
| Al-Batinah | Tom9 | 24.34^o^ N, 56.72^o^E | - | - | - | - | + |
| Al-Batinah | Tom10 | 24.34^o^ N, 56.72^o^E | - | + | - | - | - |
| Al-Batinah | Tom11 | 24.34^o^ N, 56.72^o^E | - | + | + | - | - |
| Al-Batinah | Tom12 | 24.34^o^ N, 56.72^o^E | - | + | + | - | - |
| Al-Batinah | Tom13 | 24.34^o^ N, 56.72^o^E | - | + | - | - | - |
| Al-Batinah | Tom14 | 24.34^o^ N, 56.72^o^E | - | - | + | + | - |
| Al-Batinah | Tom15 | 24.34^o^ N, 56.72^o^E | - | - | - | - | + |
| Al-Batinah | Tom16 | 24.34^o^ N, 56.72^o^E | - | + | - | - | - |
| Al-Barka | Tom17 | 23.68^o^ N, 57.90^o^E | - | - | + | - | + |
| Al-Barka | Tom18 | 23.68^o^ N, 57.90^o^E | +/+ | - | + | + | - |
| Al-Barka | Tom20 | 23.68^o^ N, 57.90^o^E | +/- | - | + | + | - |
| Al-Barka | Tom26 | 23.68^o^ N, 57.90^o^E | +/+ | + | + | + | + |
| Al-Barka | Tom27 | 23.68^o^ N, 57.90^o^E | -/+ | - | + | - | + |
| Al-Barka | Tom29 | 23.68^o^ N, 57.90^o^E | +/- | - | + | + | - |
| Al-Barka | Tom30 | 23.68^o^ N, 57.90^o^E | +/+ | + | + | + | + |
| Ibri | Tom31 | 23.23^o^ N, 56.50^o^E | +/+ | + | + | + | + |
| AES | Tom32 | 23.59^o^ N, 58.17^o^E | - | + | + | + | + |
| Al-Khoud | Tom34 | 23.56^o^ N, 58.11^o^E | - | + | + | + | + |
| Jebel Akhdar | Tom35 | 23.18^o^ N, 57.37^o^E | +/+ | + | + | + | + |
| Al-Batinah | Tom36 | 24.34^o^ N, 56.72^o^E | - | + |  | - | - |
| Al-Batinah | Tom37 | 24.34^o^ N, 56.72^o^E | - | - | + | + | - |
| Al-Batinah | Tom40 | 24.34^o^ N, 56.72^o^E | - | - | + | - | + |
| Al-Batinah | Tom41 | 24.34^o^ N, 56.72^o^E | - | + | - | - | - |
| Al-Batinah | Tom42 | 24.34^o^ N, 56.72^o^E | - | - | + | - | + |
| Al-Batinah | Tom43 | 24.34^o^ N, 56.72^o^E | - | + |  | - | - |
| Al-Batinah | Tom45 | 24.34^o^ N, 56.72^o^E | - | - | + | + | - |
| Al-Batinah | Tom46 | 24.34^o^ N, 56.72^o^E | - | - | + | - | + |
| Al-Batinah | Tom47 | 24.34^o^ N, 56.72^o^E | - | + | - | - | - |
| Al-Batinah | Tom48 | 24.34^o^ N, 56.72^o^E | - | - | + | - | + |
| AES | Tom50 | 23.59^o^ N, 58.17^o^E | +/+ | + | + | - | - |
| AES | Tom51 | 23.59^o^ N, 58.17^o^E | - | + |  | - | - |
| AES | Tom52 | 23.59^o^ N, 58.17^o^E | - | - | + | + | - |
| AES | Tom53 | 23.59^o^ N, 58.17^o^E | - | - | + | - | + |
| AES | Tom54 | 23.59^o^ N, 58.17^o^E | - | + | - | - | - |
| AES | Tom55 | 23.59^o^ N, 58.17^o^E | - | - | + | - | + |
| AES | Tom56 | 23.59^o^ N, 58.17^o^E | - | + |  | - | - |
| AES | Tom57 | 23.59^o^ N, 58.17^o^E | - | - | + | + | - |
| AES | Tom63 | 23.59^o^ N, 58.17^o^E | - | - | + | - | + |
| AES | Tom64 | 23.59^o^ N, 58.17^o^E | - | + | - | - | - |
| AES | Tom68 | 23.59^o^ N, 58.17^o^E | - | - | + | - | + |
| AES | Tom69 | 23.59^o^ N, 58.17^o^E | - | - | + | + | - |
| AES | Tom70 | 23.59^o^ N, 58.17^o^E | - | - | + | - | - |
| AES | Tom71 | 23.59^o^ N, 58.17^o^E | - | - | + | + | - |
| Khasab | Tom72 | 26.16^o^ N, 56.24^o^E | +/+ | - | + | - | - |
| Khasab | Tom73 | 26.16^o^ N, 56.24^o^E | +/- | - | + | - | - |
| Dhofar | Tom74 | 17.03^o^ N, 54.14^o^E | - | - | - | + | - |
| Dhofar | Tom75 | 17.03^o^ N, 54.14^o^E | - | - | - | + | - |
| Dhofar | Tom76 | 17.03^o^ N, 54.14^o^E | - | + | - | - | - |
| Dhofar | Tom77 | 17.03^o^ N, 54.14^o^E | +/- | - | + | + | - |
| Dhofar | Tom78 | 17.03^o^ N, 54.14^o^E | +/- | + | + | + | - |
| Dhofar | Tom79 | 17.03^o^ N, 54.14^o^E | +/- | - | + | + | - |
| Dhofar | Tom80 | 17.03^o^ N, 54.14^o^E | +/- | + | + | + | - |
| Dhofar | Tom81 | 17.03^o^ N, 54.14^o^E | +/- | - | + | + | - |
| Al-Barka | Tom82 | 23.68^o^ N, 57.90^o^E | - | + | + | - | - |
| Al-Barka | Tom83 | 23.68^o^ N, 57.90^o^E | +/- | + | + | - | - |
| Al-Barka | Tom84 | 23.68^o^ N, 57.90^o^E | +/- | - | + | + | - |
| Al-Barka | Tom85 | 23.68^o^ N, 57.90^o^E | +/- | + | + | + | + |
| Al-Barka | Tom86 | 23.68^o^ N, 57.90^o^E | +/- | + | + | + | - |
| Al-Barka | Tom87 | 23.68^o^ N, 57.90^o^E | - | - | + | + | - |
| Al-Barka | Tom88 | 23.68^o^ N, 57.90^o^E | - | - | + | - | - |
| Al-Barka | Tom89 | 23.68^o^ N, 57.90^o^E | - | - | + | + | - |
| Al-Barka | Tom90 | 23.68^o^ N, 57.90^o^E | - | - | + | - | - |
| Khasab | Tom91 | 26.16^o^ N, 56.24^o^E | - | - | + | - | + |
| Khasab | Tom92 | 26.16^o^ N, 56.24^o^E | - | + | + | - | - |
| Khasab | Tom93 | 26.16^o^ N, 56.24^o^E | - | + |  | - | - |
| Khasab | Tom94 | 26.16^o^ N, 56.24^o^E | - | - | + | + | - |
| Khasab | Tom95 | 26.16^o^ N, 56.24^o^E | - | - | + | - | + |
| Masanah | Tom96 | 23.75^o^ N, 57.63^o^E | - | + | + | - | - |
| Masanah | Tom97 | 23.75^o^ N, 57.63^o^E | - | + | + | - | - |
| Nizwa | Tom | 22.92^o^ N, 57.64^o^E | - | + | + | - | - |
| Nizwa | Tom | 22.92^o^ N, 57.64^o^E | - | - | + | + | - |
| Nizwa | Tom | 22.92^o^ N, 57.64^o^E | - | - | + | + | - |
| Sur | Tom | 22.56^o^ N, 59.51^o^E | - | - | - | + | - |
| Sur | Tom | 22.56^o^ N, 59.51^o^E | - | + | + | - | - |
