# Supplementary material for: Frequent occurrence of Mungbean yellow mosaic India virus in tomato leaf curl disease affected tomato in Oman
Source: Sci Rep. 2019 Nov 12;9:16634. doi: 10.1038/s41598-019-53106-4 (PMC6851148; doi:10.1038/s41598-019-53106-4)
Supplement: Supplementary file 2 — Supplementary Figures 1–3 [file 41598_2019_53106_MOESM2_ESM.docx]

**Frequent occurrence of *Mungbean yellow mosaic India virus* in tomato leaf curl disease affected tomato in Oman**

M.S. Shahid^1*#^, M. Shafiq^1^, M. Ilyas^2^, A. Raza^1^, M. N. Al-Sadrani^1^, A.M. Al-Sadi^1^, and R.W. Briddon^3#^

^1^Department of Crop Sciences, College of Agricultural and Marine Sciences, Sultan Qaboos University, Al-Khod 123, Oman.

^2^Cell Biology and Molecular Genetics, University of Maryland College Park, MD 20742 USA

^3^Agricultural Biotechnology Division, National Institute for Biotechnology and Genetic Engineering, Faisalabad, Pakistan.

**Supplementary Figure 1** Analysis of *Tomato yellow leaf curl virus* sequences obtained from tomato for recombination using the Recombination Detection Program (RDP). The identified recombination events are identified by coloured bars and numbered (numbers in circles) with the details of the event given in the table. The approximate positions of genes encoded by TYLCV are shown at the top of the diagram. (*) Number of methods implemented in RDP which support the event.

**
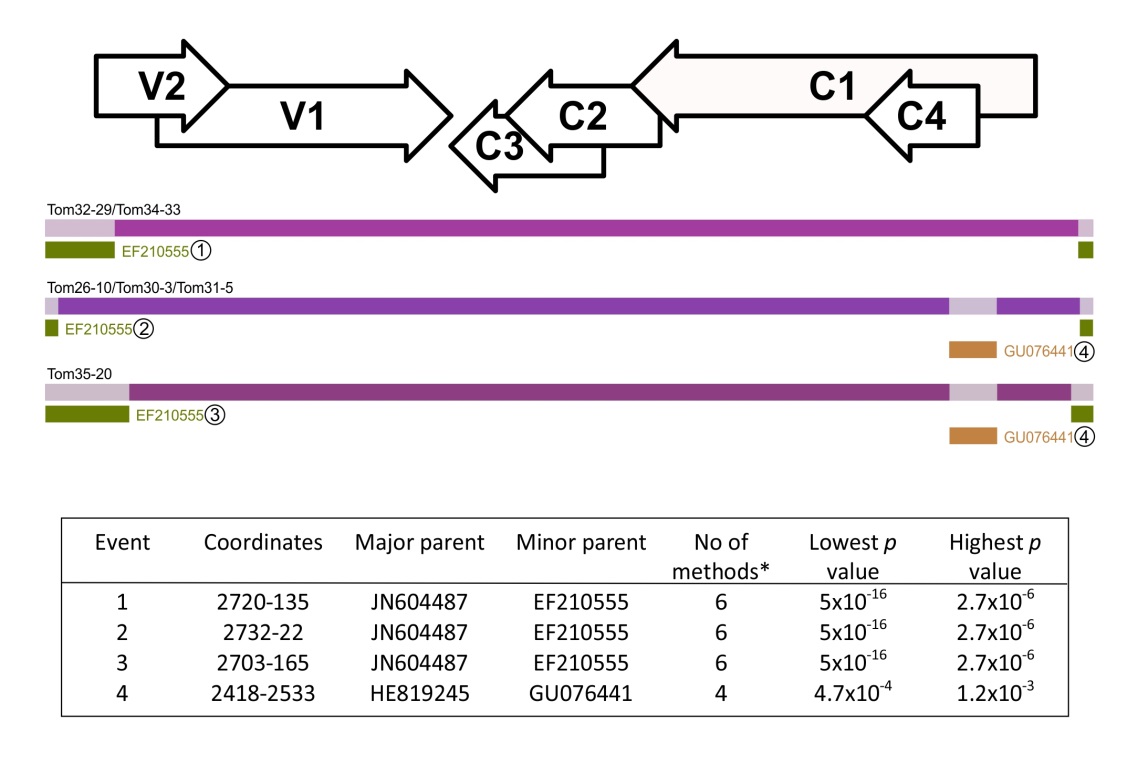
**

| **Event** | **Coordinates** | **Major parent** | **Minor parent** | **No of methods*** | **Lowest *p* value** | **Highest *p* value** |
| --- | --- | --- | --- | --- | --- | --- |
| 1 | 2720-135 | JN604487 | EF210555 | 6 | 5 ×10^-16^ | 2.7 ×10^-6^ |
| 2 | 2732-22 | JN604487 | EF210555 | 6 | 5 ×10^-16^ | 2.7 ×10^-6^ |
| 3 | 2703-165 | JN604487 | EF210555 | 6 | 5 ×10^-16^ | 2.7 ×10^-6^ |
| 4 | 2418-2533 | HE819245 | GU0776441 | 4 | 4.7 ×10^-4^ | 1. 2 ×10^-3^ |

**Supplementary Figure 2** *Mungbean yellow mosaic India virus* DNA A (A) and DNA B (B) sequences obtained from NGS of rolling circle amplified DNAs of sample Tom 26. Sequences were obtained by *de novo* assemply of NGS reads and haves been trimmed to remove duplicate sequences at the ends and to format the sequences according to accepted standards (sequence beginning from the last A in the nonanucleotide sequence [TAATATTAC])

**A**

**(2746 nt)**

ACCTGAGTGCCCCGCGACCGGTGTATTGGGGTTGCTTTAACTTTATTGCTTTTTTGGTACCCTTATCTTTTGTCGTTCAA

TCAGAAGCGCTCCTCAGCGCTATGTTAATTCAAATTTGAATTATAAAGCAAGTGGACACTATGAACCCACTAACAATGTG

GGATCCTTTGTTGAACGACTTTCCTAATTCACTGCATGGATTCCGGTGCATGTTGGCTATAAAGTATTTGCAGCAAGTTC

AAGAGAATTATCCATCTAATTCTCTTGGTTTTATGTACCTTACAGGGTTAATTCAGGTATTGCGCATCCGGAATCATGCC

AAAGCGGACTTACGATACGGCCTTCTCTACCCCGATATCGAATGCACGGAGGAGATTAAACTTCGAAACCCCGCTCCTGC

TCCCTGCATCTGCGGGAGGTGTCCCTACCAACCTGAAAAGAAGGCGATGGACCAACCGACCCATGTGGAGGAAACCTCGG

TTTTACCGACTGTATAGGTCCCCTGATGTCCCGCGGGGTTGCGAGGGACCATGTAAAGTTCAATCATTTGAGCATAGGCA

TGATATTGCTCACACAGGCAAAGTGATATGCATATCTGATGTGACTAGAGGTAATGGAATTACACATCGTCTTGGCAAAT

GATTTTGCATCAAGTCCGTGTACATTACGGGTAAGGTTTGGATGGACGAAAATATCAAGTCTAAGAATCACACAAACACT

GTGATGTTCAAGTTATGTCGTGACAGACGACCATATGGTACACCCATGGATTTTGGTCAAGTGTTCAACATGTATGATAA

CGAACCTAGTACAGCTACTGTGAAGAACGATCTGCGTGATCGTTATCAAGTCTTGCGAAGATTTAATGCCACTGTTACAG

GTGGCCAATATGCTTGTAAGGAACAAGCCATGGTGAATCGTTTTTTCAAAGTTAACAATTATGTTGTTTACAACCATCAA

GAAGCAGCGAAGTATGAAAACCATACTGAGAACGCATTATTATTGTATATGGCATGTACTCATGCCTCAAATCCTGTGTA

TGCAACGCTTAAAATTCGGATCTATTTTTATGATTCGATATTGAATTAATAAAGTTTGTATTGAACATTATGCGTAAAGC

TTACATCCTCCACCAGGTGGAGTTTTACATACAGTAAATCCGACGCGAAATATAAAATATTTGCTAAGCTAATGACACCT

ATATTATTTAGAAACCTAAATAATTGGCTACGGAAGATCGATAAGATCATCCCAGAAGTCGCCTTCATATAATGGTAGAT

CTTCAAGTCCAGGAAGCACTTGTGCATCCCCAGTGCTTTCTTCAACCCGTGGTTGAACATTATGCGTAGTTTGGTGACAA

TCATTCTGGACCCTGCTTGAATTGGACTGTGGTGCATGATCTTGAAAGAGAGGGGATTTTGCACCTCCCAGATATAGACG

CCATTCCTGAGTTGAGCTGCAGTGATGCTCTCCCCTGTGCGAAAATCCATAGTTACGGCAGTTGATATGGATGTAATAGC

TACACCCACATTTCAAGTCAATTCGAGATCGTCGAATTGCTTTCTTCTTGGCGACCCTGTGTTGAACCTTGATTGACGGA

GGAGAACAATGGTTCCTTGAGGGTGTAGAACTCCGCATTCTTTGAAGCCCACTGTTTAAGCGCAGCATTGTTCTCTTCAT

CCAAGTACTCTTTATAGGAGGACTTGGGACCAGGGTTACACAGAAAGATGGTGGGGATACCACCTTTAATTTTAGTGGGC

TTCCCGTACTTGACGTTTGATTGCCAGTCTCTTTGCGCGCCCATGAACTCTTTGAAATGTTTCAAATAATGTGGATCAAC

GTCATCAATGACGTTGTACCATGCCTCGTTAGAGTATGTTTTGTCGTTTAGATCCAAATGGCCGCAAAGATAATTATGAG

GACCTATGGCACGTGCCCACATGGTTTTACCCGTGCGACTATCTCCCTCAATAACAATACTAATAGGTCTCTCCGGCCGC

GCAGCGGGATCCCTCACATTTCTTTCAGCCCATGAAGAAATGTAGCTCGGAACCTTGTTAAATGACTCCATCGTAAAAGG

CGACTCATATGCCTGGACAGGCTCTGAGAAAATACGAGACAAATTACAATTCAAATTATGAAATTGTAAAATAAAATCTT

TAGGAGCCTTTTCTTTTAATATAAGGAGGGCCTCCAATTTCGATCCACAATTGAGTGCCTCGGCGTATGCGTCGTTGGCA

GATTGTTTACCTCCTCGAGCTGATCGGCCATCGATTTGGAAAGTTCCATGATCAAGGACGTCTCCGTCTTTCTCCATGTA

TTTTTTAACGTCTGAGCAGCTTTTAGCTGCCTGAATGTTCGGATGGTAATGTGCCGATCTGCATCTGGAATAGAGGTCGA

AGAACCTTTGGTTCCTCGTTTGTTGCTTCCCTTCGAACTGAAGCAGAACATGGAGATGAGGCTGTCCATCTTCATGAAGT

TCGCGACAGATGCGAATGAATTTCTTGTTAACAGGTGTTGACAATGCGAGAAGCTGTTCAAGAGCTTCCTCTTTTGTAAG

AGGACATTTGGGATATGTCAAGAAATAGTTTCTTGCGTTTATTGCAAAACGACCATCCCTTGGCATATTTGAAGTCGTTT

TTGTATCGGTGTACACCGATTGCTTCTCTACCCCCCTATCGGTGTATCGGTGTACTATATATACTAGAGCTACTAAAAGC

CCATAGGGGCACTCAGCTATAATATT

**B**

**(2651nt)**

ACCTGAGAGCCCCGCGACCGGTGTATCGGTATTAGAGCACGTGGGTGGTCCCTATATTACGTGGCGCGCTGTGGAGTCTC

GCTCGGAGCTTGTTTATCGAACGACTACTTGGAGTAACAGGTAACCGGATAGGTTACCGTTCGTACGTGGACAAATTCGT

CTTTTCCGTAAAAAGACCGTTATTGCCATTCGGTGTACAACTTATTATGCCTATGGCTCTTAAACCCCTTGGGGTATTTG

TGTCATTTCCCTGAAATGACCTTCTTTTTAAATCGTTTTTGGTTTATAAAAACGATCTTCGTTCAATTTGTCCACACTGT

CTTATGCGCACTATGTCGTTCGTTTATTTTAAGTTAAACTTTTCAATATCTTCATCTATATAGATGAAGGCCATGAACGT

GAGTTTCAACCATGAAATGTTTAACCGCAATTATCGCACACCATTTAAATTACGTCTTAGTAATTTTGGGTCTAGATGGC

AGCCTATGACCCCGTCAAGAGGACGTTTACGTCTTAATAAGCCTAGTGCTTCCCGTAAATTGTCATATGACCGCGTGGAA

CGGGAGATGCGTACCAATTCCATTGTTGAGGTTCAACATGGAAGCCATATGTCCCTTGAGAAGAACACGGATGTTTCTTC

ATTTGTGCAATACCCTGTTCGTGGCATCAACGGAGACGGACGTTGTAGGGATTACATCAAGTTGCTCAAACTTGATGTCT

CTGGTGTGATAAACATTAAGTCTTCGAATGGAGACCAAGACATGGAACCAGGTGACAAGTTCAGTGGCCTATTTATCCTC

ACTGTCTTGTTAGACAAGAAACCCTATCTTCCAGAAGGTGTGAACAAGTTACCCTCCTTTGCTGAGTTATTTGGACCTTA

TTCTGCTGCATATGCTAATATGCACCTCTTGGATTCTCAAAAGCCACGCTTCAAAGTCCTTGGGACAATTAAGAAGTTCG

TCAACTGCACACCTGGGACACTATATGGCCCTCTGAAATTAAATATGCCGTTGTCACGGCGAAAGTGTCCTTTGTGGACT

ACGTTCAAGGACCCTGATCAGGGTAACTGTGGTGGAAATTATAAGAATATTTCCAAAAATGCTATTGTATTGAGCTATGC

ATTTATATCAATGCATAGCCTAATTGTGGAACCATATTTTCAATTTGAATTGAAATACGTTGGATAAAGAAAATAATATT

TTATTTATTTTCATTGTGTTACAAGGCTTTGTTTACATTGGACCGTTGACTTATAAGACATTTGCTAATAGTCGTCTCTA

TAATGTCCTCTATTTCTCTCTTGCTCATTGCGTTAGAGTGGGATTGAGATATTGAATCTCCAGGATCCAATGATGCCTCT

GGCAACTTGTGCAAATGTCTAAGTGGATAATCTGCGTCGGAAGCGCTGGTGTTGTCTTGAATATTGGGCCTGTCGTTAAT

CGTGTACCGCATGGAGTTGCTGCGCCCAATAGATGACCTTGTAGCCCAAGTCTCACCTGGCTGTAGCATAATGGGCCTAT

GGCCAGTAATCGGATATGGACCCTGATTGGGTCCTGGATTAAGCATACGCCTAATGGGCTTGGGCTTCTCAACAGACCAA

AAGTCCACGCATTCTTCGTTGTAGTCCTTAGACAATATGTTTATTGTCGGGGGTTTGAATCTAATGTCTGTTGAGTGTTT

CGCGGATGACAATTTCAACTTGGCCTTTATCTGGGCAAATGTTGTTCCGTCGATCACGTTGGAGTCCTCGACTCTGTAGA

CTATCTCCCATGGTGTGTCATCCTTCAGAGAGAAGAAGGATGATGAGAAGTAATGGAGATCGACGTTACATGCAATTGGG

AAGGTGAATGCAGCTTGAGCTGCTTGTTCATAGCTTAGCCTTGTGTCACGAATTGTGACTATGACCGTTCCCTTGGCATT

AAACGGAACTTGGTTTCTGTACTCAATCACAGCATGGTCTACCTTCATGCATTTTCCCATAATCTGAACGGTCTTCTGTT

CCAGATAAGAAGGAAATTGCAACTTGATGGGCATCTCGTTGTTTGTCAGTCTGTATTCGCAACTCTTTGTCTCTACATAC

TTGTTATTAACAACTGCGCCTGAATAATTCTCCATTATTCAGAAATTGTGTTGCGAATTAATCCCTGAAAAGGAACACAC

ACCAGGAAATTATTCTATTAATTTCAAGTAGGCCGCGCAGCGGCAATTAGAGTATTTTAGATTTGTAATCATAATCTAAA

CCAAAAAGGGGTAAAAAAACTTAATACCTGATTTATTGTAATTGTTCTAGACAATTAGAGCACTAGATGTGCACAGAGGA

AGTCACAAATATTTTAGAAAATATTTGTTGTTGTCATATTTGTGATTGATTCTATCAATCGATTTAAATAAAAGACAAAC

ATGCGCAGGATTGCGTCCTGGAAATTAAATAGGGGAGTATTTAATATCCCCAAATGGATATATCTGGTGCCTCCAACGTT

TCGGTGTCTAAGCGCATGTTTCTAGAGAGAGAAGCATAGCAAAACGCAAGATGCTAGGCAAAAGAGCGTGTCGTTTCGTA

TCGGTGTACACCGATTACTTCTCTCTCTATGTATCGGTGTATTGGTGTACTATATATAGTAAAGCTACTAGGGGCTCTCA

GCTATAATATT

**Supplementary Figure 3** Three partial sequences of the DNA A component of *Watermelon chlorotic stunt virus* identified in tomato Tom 31 by NGS.

**A**

**(802 nt)**

GTACGAACAACGAGACGACGTTAAGCACACCGGTATCGTCCGGTGTGTCAGTGATGTTACTAGGGGGAGTGGAATCACTC

ATCGTGTCGGAAAAAGGTTTTGTGTGAAGTCTATATACATTCTTGGCAAGATCTGGATGGATGAGAACATAAAAAAACAA

AATCATACGAATCAGGTCATGTTCTTTCTTGTTCGTGACCGTCGTCCATATGGGTCCAGTCCAATGGACTTCGGTCAGGT

TTTTAATATGTTCGACAACGAGCCCAGCACTGCGACAGTTAAGAACGATCTTCGCGATCGTTTCCAAGTTATGCGAAAGT

TTCATGCCACCGTGGTCGGTGGCCCCTCTGGTATGAAGGAGCAGGCGCTGGTAAAACGCTTCTATCGTGTGTACAATCAT

GTGGTCTACAACCACCAAGAGTCTGCGAAGTATGAGAATCATACTGAGAATGCGATGTTATTGTATATGGCGTGTACACA

CGCATCGAATCCTGTGTATGCAACATTGAAAATTCGGATATATTTTTATGACTCAGTTACGAATTAATAAATATCGAATT

TTATTAAGTGATTTTCTTGTACATCGATCGTCCCAACAAGGACATTTTCCAATACATGACTTACGGCCCTAACAACCAAA

TTTAGACTAATTACACCTAACATATCTAGGTATTTTAGAACCTGCGTTCTAAATACCCTTAAGAAACGACCAGTCTGAGG

CCGTAAAGTCGTCCAGATTCGGAAGTTCAGGAAAGCTTTGTGAATCCCCAACGCCCTCCGAAGGTTGTGGTTGAATCGTA

TT

**B**

**(719 nt)**

CACCTGGGATATGTGAGGAAAATGTTTTTCGCTTGTATTCTAAAACGGGGAGGCCTCATGTTGACCAGTCAATTGGAGAC

ACCCCCAGATCACTAACCCCTGTATATTGGAGACTGGAGACAATATATAGAAGTAGTAAGAGGTACTACTAGGATTTTGA

CACGTAGCGGGCATCCTATAATATTACCGGATGCCCGCGGAGCCAAAAAGTGACCCCACAGGACACGTGCACCAATGAAA

TTGCGTGCTTTGAGGTAAGTTAGCTGTAAATGGAGTTTTGAAAGCGACAAGTCATACGAGTCGTATTTATAGTGCTGCTG

CGGATCAACTTTTGTCAGGATGTGGGATCCATTGCTTAATGACTTTCCCGAGTCGGTTCACGGCTTTCGGTGTATGCTAG

CTGTCAAGTACTTGCAGGCCGTTGAATCGACCTACGAGCCCAATACTTTGGGCCACGACTTGATCCGCGATCTGATTCTT

GTCCTCCGGGCCCGTGATTATGTCGAAGCGACCAGGAGATATTCTTATTTCCACTCCCGTTTCGAAGGTTCGTCGAAAAC

TGAACTTCGACAGCCCCTTCATGAGCCGTGCTCTTGCCCCCACTGTCCTCGTCACAAGCAAGCGTCGACAATGGGCCAAC

AGGCCAATGTATCGAAAGCCCAGGATGTACAGGATGTATCGAAGCCCAGATGTCCCTAAGGGCTGCGAAGGCCCATGCA

**C**

**(540 nt)**

AAACGTTCGTTGATGCCCAATCAACGAGTTCAGGCGGTAGCAACGTGAAAGACGACATCGGAAAAGGACAAACGAAAGGA

GCCACAGGTTCTTCAAAGATTCGATCCAAATTTGACTTGATATTGTGGTAGTGAAGGACGAAGTCCTTCGGTGCGAGCTC

CTTGATCACAGACATAGCCTCCCCTTTGTTACTCATGTTAAGCGCCGTGGCGTAGGCGTCGTTAGCTGTCTGTTGTCCAC

CCCTTGCAGATCGACCGTCGATTTGAAACTTGCCCCACTCGACGGTGTCGCCGTCCTTGTCCAGGTAAGACTTCACGTCT

GTACTGGACTTAGCGCTTTGTATGTTGGGGTGGAAACAGGCGCTACGGCTTGGGTGTACATGATCGAAGAGGCGATTGTC

TGTGATGGTGATCTTTCCGGCGAACTGCAGTAGCACATGTAGATGAGGTTCCCCATTCTGGTGCAGTTCTCTGCACACCT

TGACGTATTTTAGGTTCGACGGAAGGGAGAGGCCGACTAAAAAGGAAAGTAGCTCTTCTT
